# Factors Affecting Microbial Formation of Nitrate-Nitrogen in Soil and Their Effects on Fertilizer Nitrogen Use Efficiency

**DOI:** 10.1100/tsw.2001.308

**Published:** 2001-11-01

**Authors:** Alan Olness, Dian Lopez, David Archer, Jason Cordes, Colin Sweeney, Neil Mattson, Jana Rinke, W. B. Voorhees

**Affiliations:** USADA-ARS-MWA, N. Central Soil Conservation REs. Lab, Morris, MN 56267, USA

**Keywords:** clay, microbial respiration, soil organic matter, WFPS, water-filled pore space, GEMLS, general energy model for limited systems

## Abstract

Mineralization of soil organic matter is governed by predictable factors with nitrate-N as the end product. Crop production interrupts the natural balance, accelerates mineralization of N, and elevates levels of nitrate-N in soil. Six factors determine nitrate-N levels in soils: soil clay content, bulk density, organic matter content, pH, temperature, and rainfall. Maximal rates of N mineralization require an optimal level of air-filled pore space. Optimal air-filled pore space depends on soil clay content, soil organic matter content, soil bulk density, and rainfall. Pore space is partitioned into water- and air-filled space. A maximal rate of nitrate formation occurs at a pH of 6.7 and rather modest mineralization rates occur at pH 5.0 and 8.0. Predictions of the soil nitrate-N concentrations with a relative precision of 1 to 4 μg N g of soil were obtained with a computerized N fertilizer decision aid. Grain yields obtained using the N fertilizer decision aid were not measurably different from those using adjacent farmer practices, but N fertilizer use was reduced by >10%. Predicting mineralization in this manner allows optimal N applications to be determined for site-specific soil and weather conditions.

## INTRODUCTION

Each year more than 10 billion kg of N fertilizer are applied to croplands in the U.S. at a cost of more than $3.5 billion[1]. The estimated efficiency of applied N ranges from about 30% to about 70%[2]; thus, large amounts of fertilizer N are lost or wasted each year. Even on uniform soil, annual optimal application rates of applied N for maize average about 160 kg $ha^{-1}$, with a standard deviation ranging from 20 to 50%[3]. The two main factors determining applied-N use efficiency are weather and soil microbiological activity.

Nearly half (45%) of the total N fertilizer in the U.S. is applied to maize (*Zea mays* L.)[1]. Applications are often made in the autumn after harvest of the previous crop. Maize uses mainly nitrate-N ($NO_3^-$-N) and a strong correlation exists between soil $NO_3^-$-N and total N uptake or crop yield for maize and barley[4,5,6,7]. For maize, the critical $NO_3^-$-N concentration in the soil seems to be between 14 and 22 µg $g^{-1}$ in the surface 60 cm of soil, $(NO_3^-\text{-N})_{60}$[8]; this equals about 110 to 180 kg $NO_3^-$-N $ha^{-1}$.

N accumulated by the crop comes from two sources: added fertilizer or manure N and mineralization of soil organic matter and crop residues. The variation in the combined N release from these latter two sources cause large standard deviations in optimal N application rates. Fertilizer N added as ammonium-N or as urea is rapidly converted to $NO_3^-$-N; the latter form is easily leached when excessive rainfall occurs between application and maximal plant demand. Rate of mineralization of soil organic matter and crop residues varies considerably and, once converted to $NO_3^-$-N, it is also at risk of loss. A regional study in the U.S. cornbelt showed changes of +30 to –10 µg of $NO_3^-$-N $g^{-1}$ in the surface 60-cm zone within a period of about 60 days[3]. This occurred without added fertilizer N and it represents a change of between –80 and +240 kg $NO_3^-$-N $ha^{-1}$. Negative values undoubtedly represent losses via leaching and denitrification and include some immobilization of mineral N.

Three factors appear to control the rate of $NO_3^-$-N formation from crop residues and soil organic matter. These are temperature, aeration, and pH. The ability of the soil microbial community to produce $NO_3^-$-N was characterized as an N-mineralization potential ($N_o$)[9,10]. By removing $NO_3^-$-N produced in incubation vessels with periodic leaching, they showed that the total amount of $NO_3^-$-N produced eventually approached a limit. The temperature effect on the rate of N-mineralization was initially described[11] by an Arrhenius[12] type rate-coefficient.

The importance of aeration to mineralization and conversion of ammonium-N ($NH_4^+$-N) by the soil microbial community has been recognized for nearly a century[13,14]. A recent theory describes microbial respiration as a complex function of water-filled pore space (WFPS)[15,16]. Microbial respiration in soil slows if substrate diffusion (too dry) or oxygen diffusion (too wet) is inhibited.

One last effect, $[H^+]$, had been known for many years but it had been weakly described[17,18,19,20,21]. Soil pH near neutrality is optimal for promoting formation of $NO_3^-$-N and the relationship seems nearly symmetrical with respect to pH[22].

Here, our objectives were (1) development of mathematical functions describing the main factors effecting $NO_3^-$-N concentration in the soil environment; (2) assembly of these descriptions into a model predicting $NO_3^-$-N production from soil organic matter and crop residues (an N fertilizer decision aid); and (3) application of the model to field crop production.

## THEORY AND MODEL DEVELOPMENT

Respiration by the soil microbial community is the central factor in $NO_3^-$-N production of reduced N forms in either soil organic matter or fertilizer. Skopp et al.[15] attributed control of relative microbial respiration to two factors, $[O_2]$ and rate of diffusion of substrate within a specific soil. Each factor has natural limits that are directly affected by WFPS. When WFPS is minimal, $[O_2]$ approaches a maximal value but substrate diffusion approaches a minimal rate[23]. When the WFPS approaches a maximum, the rate of $O_2$ consumption exceeds the rate of $O_2$ diffusion through the system because of the continuity of the soil water. When the soil approaches saturation, the organisms resort to extraction of oxygen (electrons) from sources such as in $NO_3^-$-N, $SO_4^{2-}$-S, etc. When aeration is optimal, a maximal rate of $NO_3^-$-N production is obtained. We accept the general theory that WFPS in soil controls microbial production of $NO_3^-$-N [15,16,24].

Quadratic models give an approximation of the $NO_3^-$-N concentration over the range of conditions usually observed, but the function is symmetrical, it fails at the limits of aeration, and the $(WFPS)^2$ term is without meaningful explanation. Because the soil is a limited system, we use the general energy model for limited systems (GEMLS)[25] to describe substrate diffusion rate and $[O_2]$ effects as functions of WFPS. The GEMLS approach is empirical in that it describes the data as a function of soil moisture content without attempting to explore the mechanisms of the effect.

Data obtained by Doran et al.[16] show that while relative respiration is rather symmetrical with respect to WFPS in coarse-textured soils, a pronounced skewing is noted in finer textured soils (Fig. 1A and B). Using data published by Doran et al.[16], the water (Eq. 1) and $[O_2]$ (Eq. 2) limitation portions of the effect are described as:

$$R_{rel} = a^*(\beta + \{(e^{k(\theta-\psi)} - e^{-k(\theta-\psi)})/(e^{k(\theta-\psi)} + e^{-k(\theta-\psi)})\}) \quad (1)$$

$$+ a^*(\beta + \{(e^{-k(\theta-\varphi)} - e^{k(\theta-\varphi)})/(e^{-k(\theta-\varphi)} + e^{k(\theta-\varphi)})\}) \quad (2)$$

where $R_{rel}$ is relative respiration, a and $\alpha$ are scaling coefficients, b and $\beta$ are reference coefficients, k and $\kappa$ are coefficients of WFPS, $\theta$ is % WFPS, and $\psi$ and $\varphi$ are critical % WFPS or that value at which the rate of increase in microbial respiration begins to decline. Values of the parameters in this model are given in the Appendix. Some coefficients obviously change with soil texture and soil organic matter content, but we restrict the discussion here with a simple approximation of the relationships. Relative respiration exceeds 50% when 28% < WFPS < 85% for fine-textured soils and maximal activity occurs over a range from about 60 to 70%. For coarse-textured soils, maximal activity occurs over a rather broad range from about 45 to 70% WFPS. Clearly, the soil system is generally more sensitive to suffocation than it is to substrate diffusion, but the moisture contents must be quite large before an effect is observed. The effect of WFPS on relative respiration in volcanic soils (not shown) is sharply skewed[16], and the data are inadequate to characterize relative respiration limited by $O_2$ diffusion.

Water content of soils fluctuates between two limits. In the absence of ponding, the wettest limit or field capacity is the water content at about –33 Pa. It represents water retained against the pull of gravity. The driest state that soil achieves naturally (plant wilting point) leaves only the surface adsorbed or hygroscopic moisture remaining at about –1500 kPa. The amount of water at –1500 kPa is determined by total surface area of mineral (clay content) and organic matter.

Because soil moisture generally is retained as a function of either adsorptive or gravitational energy, the GEMLS model was used to provide an approximation of water contents at both –33 and –1500 kPa limits. Soil water content data obtained by Olson[26] for both surface and subsurface horizons was used to develop estimates of these limits. Initial fit of the GEMLS to –1500 kPa resulted in a pattern in the residual distribution and suggested a complex adsorption (Fig. 2). The two-phase model was used and the upper WFPS limit was estimated at about 56% (see the Appendix for coefficients). The large variance around the model fit indicates error in determination of texture, water content at suction equilibrium, and soil organic matter contents.

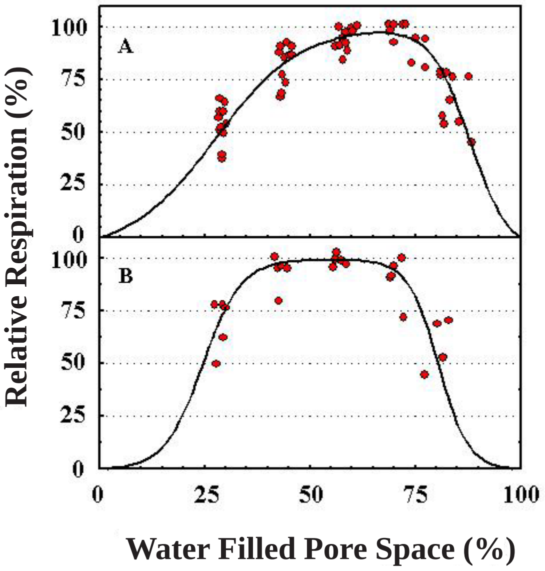

**FIGURE 1.** The effect of WFPS on relative microbial respiration for fine-textured (A) and coarse-textured (B) soils (data from Doran et al.[16]). Lines represent the sum of two supplementary GEMLS fits to the data.

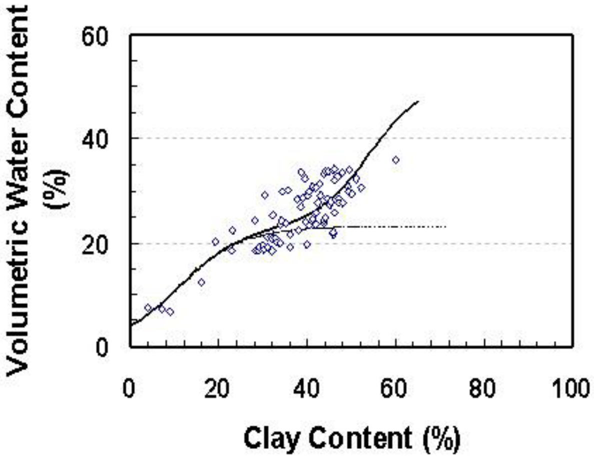

**FIGURE 2.** An example of the fit of a single-stage (dashed line) and a two-stage (solid line) GEMLS to data published by Olson[26]. Data are for –1500 kPa determinations on silt loam soils in eastern South Dakota.

Values of the stage II coefficients are estimates because no determinations were obtained at clay contents >60%. Coefficients and parameters vary with the types of clay minerals, silt, and organic matter content.

In order to estimate relative WFPS within the 0- to 15-cm depth zone of the soil, the next characteristic needed is an estimate of the total porosity. This is obtained from estimates of soil bulk density. For tilled soils, an assumption is made that tillage in the fall results in a minimal bulk density and that the soil consolidates with time. Also, that secondary tillage results in a seedbed with a bulk density of about 1.2 g $cm^{-3}$. After planting, the soil gradually consolidates to attain a maximal bulk density after harvest and this maximal bulk density is set at 1.4 g $cm^{-3}$; this assumption incorporates a slight error in the decision aid that is greater for coarse (sandy) soils than for fine-textured soils. For cropping systems without tillage, the bulk density is arbitrarily set at an equilibrium value (1.4 g $cm^{-3}$ for fine-textured soils or 1.6 g $cm^{-3}$ for coarse-textured soils). Coarse-textured soils consolidate much more rapidly than fine-textured soils but this is ignored in the decision aid. A GEMLS function is used to describe soil consolidation but, in this case, time (an abstract expression of energy) serves as a convenient substitute variable. Total energy applied to the surface in the form of rainfall, vehicular traffic, etc. should be used in place of time. The general effect of bulk density on WFPS was shown by Olness et al.[27].

A bulk density of 1.0 g $cm^{-3}$, often observed in the 0- to 15-cm zone of virgin prairie soils, is generally too porous to optimize WFPS. With this bulk density, the soil remains too dry for maximal microbial respiration. A bulk density of 1.2 g $cm^{-3}$ in the surface 15-cm depth zone, often achieved during planting operations on tilled soils, is nearly ideal for maximization of microbial respiration and production of $NO_3^-$-N in a broad range of soil textures. Without tillage of previously tilled soils, the bulk density of the 0- to 15-cm depth zone can increase to $\geq$1.4 g $cm^{-3}$ and most soils often will be too wet to support maximal microbial respiration.

The decision aid is designed to accommodate two approaches to estimation of the WFPS; an independent determination or a default value of 0.9 developed for soils that remain frozen during the winter months (December through February). During the winter, water moves from the unfrozen subsoil to the frozen surface zone[28]. Under these conditions the water content may exceed the water-holding capacity of the soil. The assumption was made that, in most cases, the soil would have about 90% of the available water holding capacity occupied shortly after thaw.

Evaporation depletes moisture and rainfall adds to the moisture content in the surface horizon. If rainfall exceeds the water-holding capacity of the surface horizon, the excess water pushes the original water with its $NO_3^-$-N to the next 15-cm increment, etc. In some cases early season $NO_3^-$-N moves below the 60-cm zone; this $NO_3^-$-N, usually a small amount, is presumed lost to crop production. Depending on soil and crop combinations, the depth of $NO_3^-$-N uptake may be expanded or contracted.

The activity of the soil microbial community, such as respiration and $NO_3^-$-N production, is quite sensitive to temperature (thermal or infrared electromagnetic energy intensity). For construction of a function for the N-fertilizer decision aid, we used data for maize[29] (*Zea mays* L.). Early growth of maize occurs below the surface of the soil and it provides a good model for the effect of temperature (see Olness et al.[25] for an illustration). It is assumed that the rate of $NO_3^-$-N production is maximized at about 30°C and that it ceases at 0°C. While the temperature limits have support in the literature (in Yeung et al.[30], for example), both the critical intensity for 50% relative activity and the relative effectiveness of a unit of thermal energy are estimates. The coefficients of the GEMLS equation are given in the Appendix.

The last major factor affecting $NO_3^-$-N production is the relative $[H^+]$ or pH. While the importance of this factor has long been recognized, the relationship had been crudely defined. Data from the USDA CSREES NCR committee 201 provided some insight into the nature of the effect of pH on $NO_3^-$-N production[8]. The data, obtained from field sites, represented production without addition of N fertilizer. At pH less than 5.0 or greater than 8.0, natural production and accumulation of $NO_3^-$-N proceeds slowly. Production of $NO_3^-$-N reached maximal values at about pH 6.7 and the distribution of $NO_3^-$-N production rates relative to pH was nearly symmetrical. This means that the apparent effect of $[H^+]$ on the rate of $NO_3^-$-N production is sharply skewed (see Olness[22] for illustration).

Again, GEMLS is applied as a two-component $[OH^-]$ and $[H^+]$ model. This implies that at least two ions are important in governing or affecting the production of $NO_3^-$-N from soil organic matter and crop residues. When this is done, maximal rates of $NO_3^-$-N production occur at pH 6.73. The parameters of the GEMLS for the effect of $[OH^-]$ and $[H^+]$ on nitrate production are given in the Appendix.

These are the functions of the variables for the main factors in natural production of $NO_3^-$-N. A final requirement is the selection of a critical soil $[NO_3^--N]_{60}$ that will optimize N fertilizer use. The user is permitted to supply a critical value. The default value is set at 20 µg $NO_3^-$-$N_{60}$ $g^{-1}$ soil. The choice of the critical value depends somewhat on the midseason climate. If optimal moisture is received, the soil microbial population will continue to produce N throughout the plant accumulation period. This is an important consideration because N accumulation is clearly divided between the vegetative and reproductive growth stages[31]. Moisture deficits realized during the reproductive growth stage of plant development curtail mineralization of N. Little is known about optimal amounts or locations of $NH_4^+$-N and $NO_3^-$-N in soil during reproductive growth.

We validated the theory by testing the N fertilizer decision aid on a range of soils in western Minnesota.

## MODEL EVALUATION

After development, the N fertilizer decision aid was tested on farms in western Minnesota during 1996 and 1998. Farmers conducted normal tillage, fertilization, and planting practices but left 6 to 12 rows of maize without added N. Each row was $\geq$0.8 km in length. Soil textures ranged from sandy loam to clay loam and pH values ranged from 5.7 to 8.2 in the 0- to 15-cm depth zone. Soil samples were collected by taxonomic mapping unit at planting time and starter N was surface broadcast at rates $\leq$50 kg $ha^{-1}$. The N fertilizer decision aid was initialized with the soil characteristics at planting time and used with current weather data to predict $[NO_3^--N]_{60}$ at the fifth-leaf stage of growth for each soil mapping unit. The fertilizer N needed for optimal economic yield was:

$$\text{Fertilizer needed, kg ha}^{-1} = [N_s - PPNT - N_m]\ \mu g\ g^{-1} * 8\ g\ \mu g^{-1}$$

where $N_s$ = a sufficient $[NO_3^--N]$ (usually about 20 µg $g^{-1}$), PPNT = the preplant soil $[NO_3^--N]_{60}$ test (µg $g^{-1}$, 0 to 60 cm), and $N_m$ = the predicted $[NO_3^--N]_{60}$ (µg $g^{-1}$) produced from preplant to five-leaf growth-stage. The factor of 8 g $\mu g^{-1}$ is an approximate conversion of concentration to mass for the 0- to 60-cm zone. Based on the predicted N need, supplemental N was applied (surface broadcast) to each soil-mapping unit. Soil samples (0 to 60 cm) were taken immediately before fertilizer application and analyzed for $NO_3^-$-N to determine relative accuracy of the decision aid. Soil bulk densities were estimated from literature sources.

Clay content was determined using the hydrometer method after $H_2O_2$ oxidation for samples obtained in 1996[32]; thereafter, values were estimated from those results. Ammonium-N and $NO_3^-$-N concentrations were determined using Cd-reduction and an AlpKem model 300 series auto-analyzer[33]. Grain yields were obtained at harvest from duplicate 30-m lengths of row from mapping units both within the test strip and adjacent areas that were fertilized by the farm operator.

## RESULTS AND DISCUSSION

Most crop production managers try to optimize a variable soil resource. A typical field usually contains two or more mapping units. The distinguishing characteristics of each unit vary both vertically and horizontally. An example of the complexity of this problem is given in Fig 3. The producer, using publicly developed advisories, applied N fertilizer before planting and achieved a generally desirable result in Fig. 3A. However, due to postplanting weather events, about half of the applied N apparently was lost due to leaching and runoff. The consequence of this situation was a grain yield that mirrored the soil $[NO_3^–\text{-}N]_{60}$ obtained in June (Fig. 3B) and a substantial loss of N fertilizer and grain yield potential.

When management is such that the crop accumulates most of the $NO_3^–$-N produced and the residual or carryover concentration is small, differences in $[NO_3^–\text{-}N]_{60}$ across the landscape are usually trivial and generally can be ignored as factors in environmental risk or production economics. However, when excess N fertilizer has been applied or weather conditions restrict uptake of N, residual concentrations can be large and variation within the field, if ignored, can lead to leaching losses or diminished N fertilizer use efficiency.

Except for one location, fertilized the previous fall, initial $[NO_3^–\text{-}N]_{60}$ verified that the fields had not been fertilized. For all sites, initial spring $[NO_3^–\text{-}N]_{60}$ ranged from a mere 1.5 µg $g^{–1}$ on a silt loam soil to 24.2 µg $g^{–1}$ on a fine-textured clay loam that had been fertilized in the previous fall (Table 1). Total $[NO_3^–\text{-}N]_{60}$ ranged from about 12 to 200 kg $ha^{–1}$, if soil bulk densities were in the typical range of 1.2 to 1.4 Mg $m^{–3}$. More importantly, $[NO_3^–\text{-}N]_{60}$ often varied within a field by 50 to 100% depending on the soil characteristics. Starter fertilizer, 30 to 50 kg N $ha^{–1}$, was broadcast on the test strips at planting because at least this amount of fertilizer N would be needed to meet the potential crop demand. In Table 1, the change in $[NO_3^–\text{-}N]_{60}$ is reported with and without adjustment for the starter fertilizer N. The adjusted values assume that (1) all starter fertilizer was retained on the field, and (2) that subsequent sampling recovered all of the applied N. While both assumptions in calculation of the adjusted estimates are unlikely, they represent the extreme limit of fertilizer N recovery. The average change in $[NO_3^–\text{-}N]_{60}$ within a field ranged from –1.37 to 4.27 µg $g^{–1}$ (adjusted) or –1.29 to 9.62 µg $g^{–1}$ (unadjusted). With the exception of sites 4, 6, and maybe 7 the predicted change was usually intermediate to the adjusted and unadjusted values. Observations at sites 4 and 6 were from similarly fine-textured soils that are expected to be sensitive to errors in estimates of soil moisture content or rainfall received.

With the exception of site 6, the N fertilizer decision aid predicted changes in the $[NO_3^–\text{-}N]_{60}$ on average within 2 to 4 µg $g^{–1}$ (Table 1). More importantly, grain yields obtained using the decision aid were on average not measurably different from those with farmer fertilizer applications with the exception of those from site 4; here, use of the decision aid markedly improved mean yields. In site 4, only those sites with predicted $[NO_3^–\text{-}N]_{60}$ <20 µg $g^{–1}$ and to which fertilizer N was added were evaluated. Those soils within site 4 had either lost fertilizer N or the mineralization of soil organic matter was measurably retarded in comparison to the remainder of the field.

In most cases mean soil $[NO_3^–\text{-}N]_{60}$ increased during the 30 to 60 days between samplings. Within a field, differences between observed and predicted changes in $[NO_3^–\text{-}N]_{60}$ between sites varied from <1 to >10 µg $g^{–1}$. Part of the explanation for the large variance lies with the simplifying assumptions made in assembling the decision aid. Among the more important assumptions is that of infiltration of a constant fraction of rainfall. Surface depressions, areas in which runoff collects and infiltrates, are ignored. This assumption had little effect on the results obtained

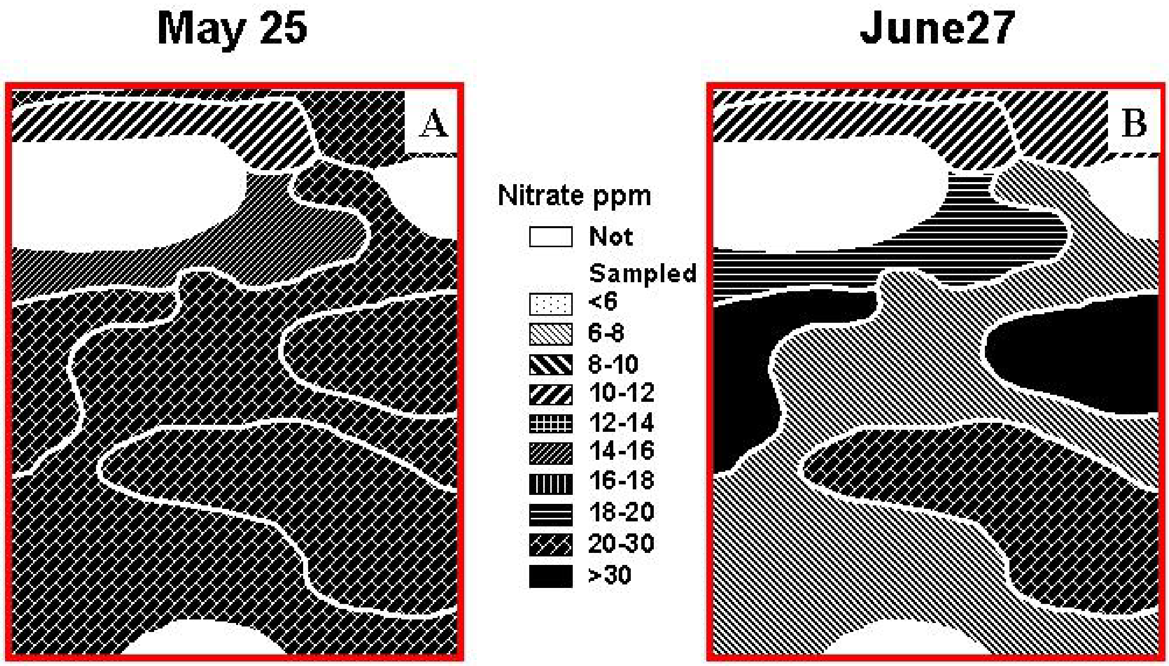

**FIGURE 3.** The $[NO_3^–\text{-}N]_{15}$ for a 33 ha field in western Minnesota on May 25 (A) and June 27 (B). Intense rainfall in early June leached much of the $NO_3^–$-N from large portions of the field. Lines represent soil mapping unit boundaries.

**TABLE 1**
**Average Observed and Predicted Changes in Soil $[NO_3^{-}\text{-N}]_{60}$***

| Site | $[NO_3^{-}\text{-N}]_{60}$ Planting | Δ $[NO_3^{-}\text{-N}]_{60}$ by 5th to 7th Leaf Growth Stage: Unadjusted* | Adjusted** | Predicted§ | *n* |
|---|---|---|---|---|---|
| | µg g$^{-1}$ | | | | |
| 1 | 6.09 ± 0.65 | 2.36 ± 2.68 | –1.37 ± 2.68 | 1.36 ± 1.29 | 6 |
| 2 | 5.08 ± 1.21 | 2.84 ± 0.89 | –0.68 ± 0.89 | 1.79 ± 1.41 | 8 |
| 3 | 6.73 ± 2.53 | 7.40 ± 2.23 | 1.73 ± 2.23 | 4.90 ± 3.73 | 6 |
| 4 | 19.3 ± 4.32 | –1.29 ± 4.84 | — | 2.45 ± 1.49 | 9 |
| 5 | 6.53 ± 1.89 | 5.57 ± 5.38 | 0.12 ± 5.38 | 4.05 ± 5.54 | 5 |
| 6 | 9.79 ± 3.05 | 9.62 ± 6.58 | 4.27 ± 6.58 | –2.52 ± 4.52 | 7 |
| 7 | 2.34 ± 1.01 | 3.05 ± 1.39 | –1.24 ± 1.38 | 3.12 ± 1.44 | 10 |

* Values represent means ± standard deviations.
** Observed change without consideration of broadcast fertilizer applied after initial sampling.
§ Calculated change assuming total conservation of fertilizer applied after initial sampling and complete recovery in the second sampling.

even for sites 2 and 7; slopes exceeded 3% and some soils accommodated water initially received on others. The error in water for these two sites seems to have had an immeasurable effect on the predicted $[NO_3^{-}\text{-N}]_{60}$. The decision aid also ignores antecedent rainfall effects in the partitioning of water between infiltration and runoff.

In spite of the fact that use of grain yield as an assessment measure has limitations, the yields from the test strips were on average not measurably different from those managed by the producers. In site 5, a late frost caused a 50% yield reduction in affected areas but these areas were irregular in shape and location. Affected plants produced very small ears with little grain and failed to develop a generally robust growth. When the affected comparisons are ignored, the test results show agreement (data not shown). In site 2, midseason drought at flowering severely affected a few sites but both test and producer plots suffered about the same degree of damage.

Antecedent crop is recognized as a factor in current crop performance; soybean ([*Glycine max* L.] Merr.) had been grown at most sites the previous year. Soil tillage method also affects soil temperature through disposition of surface crop residues. All sites had been tilled using a chisel- or moldboard plow in the previous fall except for the half of those on site 7 for which no tillage was conducted.

Producers tend to apply N fertilizer based on knowledge of the individual resource and an integration of economics averaged over years. No validation of the amounts of fertilizer N applied by the producers was available. Their individual estimates are based on total amounts of N purchased and the areas on which the N was applied. In spite of the lack of quantitative measure, however, N fertilizer applications, based on spring testing and decision aid projections of need, were less than producer applications by >10 %. Reductions in N fertilizer use were probably due to the fact that weather conditions favored efficient mineralization of soil N; under less favorable weather conditions, the decision aid could conceivably lead to greater rates of application of N fertilizer than traditional producer application rates.

## CONCLUSIONS

Variation in N-fertilizer use efficiency is partly due to variation in the amounts of $NO_3^{-}$-N produced by microorganisms from plant residues and soil organic matter as well as residual N from the previous season. Amounts of microbially produced N vary considerably from year to year but reasonable predictions of this N can be made from consideration of physical and chemical characteristics of the soil and weather factors. The main weather factors are rainfall and temperature and the main soil factors are texture, pH, and bulk density. Coupling computerized decision aid technology with precision application of N minimizes waste and risk of degradation of the soil and water resources used to produce food and fiber.

## ACKNOWLEDGEMENTS

The authors gratefully acknowledge the assistance of farmers in western Minnesota as well as the staffs at the University of Minnesota-Morris and the USDA-ARS North Central Soil Conservation Res. Lab. Especially noteworthy was field assistance by L. (Skip) Maanum and Charles Hennen and laboratory analyses by Jay Hanson and Hope Stoller. We also gratefully acknowledge the manuscript reviews provided by Dr. T. Schumacher, South Dakota State University, and Dr. M. Russelle, USDA-ARS at the University of Minnesota.

| | | |
|---|---|---|
| Received: | June | 25, 2001 |
| Revised: | October | 5, 2001 |
| Accepted: | October | 15, 2001 |
| Published: | November | 1, 2001 |

**APPENDIX 1**
**Values of GEMLS Parameters**

| Factor | Coefficient | | | Critical | Coefficient | | | Critical |
|---|---|---|---|---|---|---|---|---|
| | Scaling a | Reference b | Energy k | Energy $\psi$ | Scaling $\alpha$ | Reference $\beta$ | Energy $\kappa$ | Energy $\varphi$ |
| WFPS | | | | | | | | |
| Loam-Clay | | | | | | | | |
| Loam | 53.60 | –0.05 | 0.05 | 27.45 | 53.75 | –0.08 | 0.11 | 87.88 |
| Sand-Sandy | | | | | | | | |
| Loam | 50.00 | 0.00 | 0.11 | 24.50 | 50.0 | –0.01 | 0.14 | 80.49 |
| Volumetric | | | | | | | | |
| water content | | | | | | | | |
| –33 kPa | | | | | | | | |
| without C | 17.00 | | 0.0725 | 12 | 9.1 | | 0.04 | 58.06 |
| with C | 18.48 | | 0.12 | 0.9 | 8.75 | | 0.09 | 51.527 |
| –1500 kPa | | | | | | | | |
| without C | 11.57 | | 0.07 | 11 | 15.7 | | 0.08 | 56 |
| with C | 12.5 | | 0.10225 | 0.85 | 14.8 | | 0.08 | 56 |
| Temperature | | | | | | | | |
| T (°C) | 1.5 | 1.0 | 0.142 | 19.7 | | | | |
| pH* | | | | | | | | |
| $[H^+]$, $[OH^-]$ | 14 | 0.0 | 0.8 | 1.05 | –14 | 0.3 | 7.0 | 0.24 |

* From Olness[22].
